# Beyond the three monkeys of workforce diversity: Who hears, sees, and speaks up?

**DOI:** 10.3389/fpsyg.2022.879862

**Published:** 2022-09-15

**Authors:** Fatma Kusku, Ozlem Araci, Veysi Tanriverdi, Mustafa F. Ozbilgin

**Affiliations:** ^1^Department of Management Engineering, Istanbul Technical University, Istanbul, Turkey; ^2^School of Organizations, Systems and People, University of Portsmouth, Portsmouth, United Kingdom; ^3^Department of Psychology, Zonguldak Bülent Ecevit University, Zonguldak, Turkey; ^4^Brunel Business School, Brunel University London, London, United Kingdom

**Keywords:** workforce diversity, diversity management, diversity works, social identity, human resource management, Turkey, belonging, otherness

## Abstract

The purpose of this study was to explain differences between employees who feel a sense of belonging and those who feel a sense of otherness in terms of their opinions about diversity works in their organizations. We conducted an empirical study to examine the perceptual differences between two independent groups of the study “who feel a sense of belonging” and “who feel a sense of otherness.” We collected data from 792 employees working for organizations in different sizes, industries, and capital structures, which enriched the representativeness of the sample. The findings show that out-group members remain less satisfied with diversity works in their organizations based on four main issues such as “competence of diversity actors,” “embeddedness of diversity works in organizational policies/practices,” “diversity awareness in the HRM functions,” and “diversity-related employee satisfaction.” This paper makes two contributions. First, it contributes to the extant literature an understanding of the differences between those who remain indifferent to diversity works and those who care to see, speak, and hear about them. Second, with a few exceptions, extant studies on diversity works have been dominated by Western-centered research. Research is needed on countries with different macro-contextual conditions, such as different legal regulations, socio-political status, and history. For this study, survey data were collected from people who work in Turkey, a country which has limited legal measures and underdeveloped discourses for equality, diversity, and inclusion. The paper provides significant insights into leading diversity works in national settings with less developed supportive mechanisms for diversity.

## Introduction

A recent report by the Organisation for Economic Co-operation and Development (OECD, 2020) demonstrated challenges to a broader recognition of diversity works (policies, practices, and interventions of workforce diversity), one of the most significant of which appears to be the indifference of individuals to diversity issues and concerns. Using the metaphor of three monkeys, which refers to the maxim “see no evil, hear no evil, speak no evil,” which signals remaining indifferent, we question whether it is the individuals among those employees with a sense of belonging or otherness are the ones who transcend such indifference to diversity works. We define diversity works as all efforts at the organizational level to engage with, plan, and manage workforce diversity. This paper explores who remains indifferent to diversity works and who cares to see, speak, and hear about them. To query this interesting phenomenon, we turn to social identity approaches, which explore in-group and out-group behaviors (see Tajfel and Turner, 1979; Turner et al., 1987). According to these approaches, cognitive processes and behaviors change based on group identity. We query whether there are differences between in-group (the group to which the person feels they belong, having similar characteristics) and out-group (a group to which the person does not feel belonging, having different characteristics) members in terms of their perceptions of diversity works. So, we question whether feeling a sense of belonging (feeling or experience of having similar characteristics) and otherness (feeling or experience of having different characteristics) (Özbilgin and Woodward, 2004) in the organization explains indifference to diversity works. As indifference presents a significant barrier to effective implementation of diversity works, it is essential to understand how in-group (who feel a sense of belonging) and out-group (who feel a sense of otherness) members respond to diversity works. Such an understanding of different attitudes toward diversity can offer organizations ways to craft their diversity works to cater to the divergent needs and responses of in-group and out-group members.

Human resource management (HRM) often takes on the policies and practices that regulate the relationship between the organization and employees (Gilbert et al., 1999; D’Netto et al., 2014; Sabharwal, 2014; Berger et al., 2016). In countries where diversity management has not developed a unique professional identity, the HRM often takes on the role of managing diversity. Benschop (2001) explained that there is a tendency to treat employees as a homogeneous group whose differences are ignored in the HRM activities. Worse still, the unitarist approach to the HRM even assumes that the interests of employees and employers are aligned (Geare et al., 2006). However, the perceptions, thoughts, feelings, and behaviors of people who see themselves as part of the established order in the organization and those who feel a sense of otherness due to their particular characteristics may be different (Allen, 2010). The HRM function is uniquely positioned to have insights into employees’ sense of belonging and otherness through its access to employee surveys and professional practice.

This study has two major contributions to the literature on diversity works. First, this research contributes to the extant literature an understanding of the differences between those who remain indifferent to diversity works and those who care to see, speak, and hear about them. The literature on the antecedents, correlates, and consequences of diversity works views the relationship between diversity and organizational outcomes as complex and multifaceted (e.g., Shore et al., 2009). Evidence from the studies shows that workforce diversity on its own has both negative and positive consequences (see Yadav and Lenka, 2020). What makes the difference is the effective management of diversity if the organizations wish to accrue the benefits of diversity (Özbilgin et al., 2016). This study shows how employees respond to diversity works based on their position in the axis of belonging and otherness at work.

Second, with a few exceptions, extant studies on diversity works have been dominated by Western-centered research (see Karsten et al., 2011). Because the assumptions of managerial concepts developed in industrialized countries may not be directly transferred to peripheral contexts due to differences in the corporate environment (Erçek, 2016), the assumptions and findings of research conducted in Western-centered countries cannot be universally accepted (Klarsfeld et al., 2019). Therefore, research is needed on countries with different macro-contextual conditions, such as their legal regulations, socio-political status, and history. For this study, survey data were collected from people working in Turkey, an underrepresented context that provides a unique setting to explore diversity works with both Western and non-Western characteristics (Erdur, 2022). Populist and negative reactions to diversity works are rampant internationally (Vassilopoulou et al., 2016; Saba et al., 2021). As a country, which has limited legal measures, and underdeveloped discourses for equality, diversity, and inclusion (Özbilgin and Yalkin, 2019; Kornau et al., 2021; Küskü et al., 2021), Turkey has an increasingly diverse workforce and offers an important context in which to study how individuals with either a sense of belonging or a sense of otherness respond to the limited provision of diversity works. We particularly problematize the indifference of those with a sense of belonging to diversity works in the Turkish context.

### Theoretical background, literature review, and hypotheses

#### Conceptual background from the perspective of social identity

According to the research, individuals are generally more attracted to those similar to themselves, and they tend to communicate better with them. As a reflection of this, they work more harmoniously (O’Reilly et al., 1989), experience less relationship conflict (Jehn et al., 1999), have a lower turnover rate, and feel more loyalty to their organizations (Tsui et al., 1992). On the contrary, people are less attracted to “others” who are not like themselves and they display difficulty in communicating with dissimilar others (Triandis, 1959), and this experience of difference creates a negative reinforcement in relationships (Martins et al., 2003). In other words, individuals do not trust those they see as different from themselves (Blalock, 1967). Moreover, as the workforce diversity increases over social identity categories such as gender, race, ethnicity, and age, some psychological barriers may arise between the social interactions of different groups (Blalock, 1967; Kamasak et al., 2019). These barriers can be a significant threat that can lead to other problems within the organization. Therefore, according to the principle of social similarity, it is stated that when people are given a free choice, they tend to work and establish relationships with people who are similar to them (Tsui and O’Reilly, 1989; Martins et al., 2003). Of course, organizations can choose to let or not let workforce diversity enter the organization for various reasons (see Ely and Thomas, 2001). However, economic, political, and social developments trigger heterogeneity and greater diversity. In this case, a lack of competencies to understand and craft diversity works causes problems. Ignoring diversity and individual differences in business and management can pose a serious threat to individual rights and the social order and harmony at work. Therefore, organizations try to engage in diversity works to overcome diversity challenges and accrue organizational benefits.

Managing diversity is complicated by social identities, which color individuals’ judgment of their competencies compared with their in-group and out-group members (e.g., O’Reilly et al., 1989). Therefore, we think that the social identity approach, which explores in-group and out-group perceptions and behaviors, offers a helpful theoretical lens through which we can examine the differences between people who feel a sense of belonging and those who feel a sense of otherness in terms of their perceptions of diversity work issues at work. Social identity approaches imply that perceptions, thoughts, and behaviors of one’s personal identity are different from one’s social identity, which emerges in their association with a social group (Tajfel and Turner, 1979; Turner et al., 1987). This means that differences in opinions come to the fore, especially in certain contexts where individuals define themselves through belonging to a group. In other words, social divisions occur in situations where individuals are influenced by their social identities. When people perceive themselves as members of a group, they simultaneously think and act differently from their personal identities (Avanzi et al., 2021). While individuals are likely to favor members of their in-groups above and beyond members of out-groups in their social interactions, the formation of social identity along with gender, ethnicity, or other demographic lines could lead to negative consequences such as ethnocentrism, mutual influence, shared norms, groupthink, normative behavior, emotional contagion, and stereotyping (Hogg and Terry, 2000).

This process also runs in organizational contexts. Identifying themselves with a social group (e.g., gender, age, ethnicity, etc.), people can classify themselves with their career (personal level), with their team or department (group level), or with their whole organization as a specific form of social identification (Ashforth and Mael, 1989; Van Dick and Wagner, 2002). This kind of self-categorization causes depersonalization in which individuals represent the relevant group prototype above and beyond their personal identity (Hogg and Terry, 2000). The main reason for this situation is the motivation to get rid of uncertainty and to have positive self-esteem by gaining a place in reality. Since having a positive self-esteem derives partly from individuals’ social identity, people tend to attribute more positive traits to their in-group than out-group (Tajfel and Turner, 1979). In other words, people must perceive their in-group as superior to out-group to feel better.

Consequently, this situation causes indifference to injustices against the out-group members and discrimination against them (Tajfel et al., 1971). This is particularly important for our study, as diversity works attempt to remedy the historical inequalities that haunt workforces. Individuals from historically disenfranchised groups such as women and minority ethnic groups are likely to be more interested in what diversity works could offer them. However, individuals from dominant in-groups in society are more likely to be disinterested in diversity works.

How identification affects job satisfaction, job involvement, extra-role behaviors, and turnover intention can be seen in some meta-analyses (Riketta, 2005; Lee et al., 2015; Steffens et al., 2017). However, such studies are not concerned with belonging and otherness and concern for or indifference to diversity works. In brief, individuals’ perception is influenced by their social identities in the way that individuals act in the interests of their groups, do not accept criticism from their group, consciously or unconsciously hold their group members in a more advantageous position, and do not see the injustices done to others (Mergen and Ozbilgin, 2021). Therefore, we think that when the context is diversity works, the perception of those who feel a sense of belonging and otherness may be somewhat different. We examined two salient aspects of the social identity approach, i.e., in-group (with a sense of belonging) and out-group (with a sense of otherness) responses to diversity works across the following four dimensions: (1) opinions on the competence of diversity actors, (2) opinions on the embeddedness of diversity works into the organizational policies/practices, (3) opinions on diversity awareness in the HRM activities, and (4) opinions on diversity-related employee satisfaction. We selected these dimensions to cover employees’ opinions, observations, experiences, and perceptions regarding organizational attempts to manage diversity. Even though categories such as gender, ethnicity, and disability still dominate diversity works (see Sinicropi and Cortese, 2021; Triana et al., 2021), because of universally accepted human rights values, diversity works are more profound when it considers the intersections between diverse groups and power relations (Tatli and Özbilgin, 2012; Köllen, 2021). Therefore, in the case of our paper, we examine the feelings of belonging and otherness, rather than picking any particular category of diversity, to mobilize the social identity approach.

#### Opinions on the competence of diversity actors

The relevant competence of diversity actors, who assume responsibility for implementing diversity-related activities, is a necessary condition for the effective implementation of diversity interventions (Riccò and Guerci, 2014; Carstens and De Kock, 2017; Dang et al., 2022). The support, pioneering, and leadership of the senior management, as significant diversity actors, are considered prerequisites for the successful implementation of practices within the scope of diversity management in an organization (Herrera et al., 2011; Ng et al., 2020). If the senior management cannot be sufficiently competent and well versed in managing diversity, the scope of activities to be carried out in this context (Ng and Burke, 2005; Dang et al., 2022) and the level of adoption and acceptance of the activities attempted to be carried out would be low (Ng and Wyrick, 2011; Carrillo Arciniega, 2021). It is essential to examine whether there are any differences in opinion about the competence of diversity actors between in-group and out-group members.

On the contrary, although the most significant responsibility for paying attention to diversity within the organization and the implementation of the programs created within this scope belongs to the senior management, for this understanding to spread within the organization, both HRM unit managers and other managers must assume responsibility (Pitts et al., 2010; Riccò and Guerci, 2014; Mullins, 2018). To manage diversity effectively, all employees in the organization should be included in the process, and they should carry out the operation together (Kalev et al., 2006; Shen et al., 2009; Palalar Alkan et al., 2022). If this can be achieved, the organization’s strategic-, tactical-, and operational-level practices could be harmonized to achieve diversity goals, which may lead to both an increase in the scope of diversity management and an increase in the benefit obtained from the activities. If so, to increase the practices within the range of diversity works within the organization, especially the groups responsible for this should have enough knowledge of workforce diversity and its management. As the knowledge level of the responsible persons on the subject increases, it will be easier to understand the relevant diversity issues among employees. Consequently, the scope of the diversity factors taken into account in organizational practices will naturally expand. According to social identity approaches, individuals do not accept the negative evaluations of their group because these evaluations of their group are perceived as their own. Relatedly, it can be said that even if the diversity actors would not be competent enough, those who feel a sense of belonging will perceive them as competent. Therefore, we can predict that individuals who feel a sense of belonging would be less likely to problematize the competence of the diversity actors, as their interests would be aligned with those of their organizations. Therefore, in Hypothesis 1, we question whether the people who feel a sense of belonging and those who feel a sense of otherness have different perceptions of the competence of diversity actors at work.

*H1*: Those who feel a sense of belonging will perceive their diversity actors as more competent than those who do not.

#### Opinions on the embeddedness of diversity works in organizational policies/practices

Although some relationships are mentioned (e.g., Ely and Thomas, 2001; Point and Singh, 2003; Ward et al., 2022), there is no clarity about the causal relationship between accepting, adopting, and managing diversity and individual, group, and organizational benefits (Kellough and Naff, 2004; Pitts et al., 2010; Yadav and Lenka, 2020). But simply complying with established quotas and government regulations does not mean managing diversity effectively (Syed and Ozbilgin, 2019). Accepting and managing diversity within the organization is, in a sense, a significant effort to imagine and support a workplace that recognizes and accommodates differences (Friday and Friday, 2003; Acar, 2010; Berrey, 2014). For this reason, for diversity to be accepted within the organization and to make the necessary arrangements, diversity considerations must be systematically incorporated into the organizational strategies and general policies (Davis et al., 2016; Calvard, 2020). Additionally, fostering a climate of inclusion is essential to achieve diversity goals (Mor Barak et al., 2016). Although developments in this area differ according to institutional contexts, studies (see Riccò and Guerci, 2014) state that activities within the scope of managing diversity are often managed without realizing that they should be a part of the organization’s basic strategies and policies. Unless it is transformed into a part of the organization’s strategy and policies, it will not be easy to develop the activities carried out within the scope of managing the diversity in the workforce to meet the expectations of all parties. Social identity approaches posit that individuals who feel a sense of belonging in organizations are more likely to have their interests aligned with the strategies and practices of their organizations.

*H2*: Those who feel a sense of belonging will be more likely to perceive that diversity considerations in their organization are embedded into the organizational policies/practices than those who do not.

#### Opinions on diversity awareness in the human resource management function

Working together with people with different characteristics in certain respects is not a guarantee of effective management of diversity (Sartori et al., 2022). It is necessary to create supportive cultures that will consider all stakeholders’ concerns, including employees from underrepresented backgrounds (Nadarajah et al., 2022). For this to be achieved, there is a need for management practices (Ehrke et al., 2014; Roberson, 2019) and inclusive functions for all employees (Geiger and Jordan, 2014; Roberson and Perry, 2021) that can make people work together in harmony. In many studies (e.g., Pitts, 2006; D’Netto et al., 2014; Berger et al., 2016), it is stated that diversity can only be managed through the HRM activities of the organization. According to some studies, organizations that became aware of this situation started to change their HRM practices by the end of the 1990s (Kemper et al., 2016) and diversity management has become an increasingly important part of HRM in organizations (Davis et al., 2016). Indeed, workforce diversity is one of the main challenges in HRM (Showkat and Misra, 2022).

For this reason, examining diversity awareness in the HRM functions is relevant. To keep justice and impartiality, to be liable to the needs of different people, and to offer equal opportunities through the HRM functions are critical to see the commitment of HRM departments to diversity. Reflecting on the idea of social identity approaches, individuals act to keep their group interests superior to those of the other group to perceive their group in a better position to maintain a positive self-image. Conversely, they behave in such a way that keeps other group members in a disadvantaged position (Tajfel et al., 1971; Schneider and Northcraft, 1999; Kramar and Jepsen, 2021), so that we can predict that individuals who feel a sense of belonging at work would not find the diversity awareness of the HRM function inadequate as they would not view diversity interventions as relevant or necessary for their belonging at work. We formed Hypothesis 3 to understand the difference between the employees’ opinions on the issues of fair and impartial conduct of the processes carried out in organizations within the scope of HRM, creating sensitivity to differences and realizing them in a way that ensures equal opportunities.

*H3*: Those who feel a sense of belonging will be more likely to perceive that there is diversity awareness in the HRM functions in terms of (H3a) justice and impartiality, (H3b) sensitivity to the needs of people from diverse backgrounds, and (H3c) equal opportunity than those who do not.

#### Opinions on diversity-related employee satisfaction

When employees’ individual identities are salient and they identify with their career, their behaviors and attitudes will be influenced by personal characteristics. On the contrary, if an employee perceives themselves as a member of a team, department, or organization (when the group-team identity is salient), their behaviors and attitudes will be affected by those social ties (Ashforth and Mael, 1989). There are findings that diversity reduces job satisfaction (e.g., Choi, 2013) and employee satisfaction, which refers to the extent to which employees are satisfied with their job in general or the specific facets of the job within the organization (Dineen et al., 2007), and hurts the psychology of employees in general (Taras et al., 2019). However, as highlighted in studies examining the positive relationship between diversity climate, diversity training, trust, and turnover (e.g., Ward et al., 2022, Zhang and McGuire, 2022), the adverse effects of diversity can be compensated for if managed (Stazyk et al., 2012; Showkat and Misra, 2022) and employee satisfaction levels can increase (Pitts, 2009); minority employees may accrue benefits from diversity management activities (Pitts, 2009; Ward et al., 2022). Some studies (e.g., Memon et al., 2021) indicate that the level of employee satisfaction with the HRM practices has a significant effect on the overall satisfaction level of employees.

According to current research, people’s experiences with events shape their responses within the organization (see Bond and Haynes, 2014). In this case, employees (those who feel a sense of otherness) who indicate dissatisfaction with the HRM practices in terms of managing diversities, that is, those who have low levels of satisfaction in this regard, will naturally have low levels of overall satisfaction. However, there are studies that have found that the satisfaction levels of different groups among employees will be different (e.g., Tsui et al., 1992; Küskü, 2001, 2003), and the availability of studies that emphasize the satisfaction level of those who feel a sense of belonging and otherness is restricted. Social identity approaches imply that when an individual perceives themselves as a member of an organization, they will get a high level of satisfaction because satisfaction is more likely associated with the relationship with the other members of the group (Leach et al., 2008). The more individuals identify with their organization, the more personnel are satisfied and the more job satisfaction (Van Dick et al., 2006). Therefore, it made sense to us to construct Hypothesis 4.

*H4*: Those who feel a sense of belonging will show a higher score of diversity-related satisfaction than those who do not.

## Methodology

### Procedures, sample, and data collection

Diversity management practices vary across countries and organizations (Kellough and Naff, 2004; Bacouel-Jentjens and Yang, 2019). Differences in social concerns and environmental dynamics of the countries should be considered when using the same measurement tool in different countries. Ignorance of contextual differences may result in misleading results. In this study, we have paid attention to using measurement tools appropriate for the research context. For this purpose, we investigated relevant literature and conducted interviews to ensure the contextual appropriateness of the measurement tool.

Data collection through questionnaires is one of the sources of research challenges. “Linguistic, contextual misunderstandings, and respondent carelessness” are potential problem creators that are difficult to determine and correct their effects by statistical analysis (Einola and Alvesson, 2021). To deal with these challenges, we conducted a pre-study to examine and minimize the gap between what we are trying to collect and what respondents attribute meaning to the questions. Doing pre-study mitigates some of the problems that are produced by a gap between “the mind of the researcher” and “the mind of the respondents” (Einola and Alvesson, 2021, p. 3). After testing the functionality of the questionnaire with a pre-study, we made the necessary corrections to the form and moved to the data collection stage.

We collected data from the following two channels to increase the representativeness of the sample: (1) members of the alumni association of the university where the first researcher works and whose graduates are distributed in all cities of the country and (2) Linkedin connections of researchers. To understand whether there is a difference between the data coming from two different channels, the data were collected over two different online systems. Because there was no statistically significant difference between the data collected through different systems, all incoming data were combined and included in the analysis. We sent messages to potential participants that have the purpose of the study, ethical assurances, and the web address of the questionnaire. In this message, we also requested them to share our announcement message with their professional networks to increase the number of participants. We sent our announcement messages ten days after the first invitation.

To deal with the problem of social desirability (Spector and Brannick, 2009) as a result of the usage of the self-administered questionnaire method (Babin and Zikmund, 2015), the participants were informed that the collected data would be used only for scientific purposes in the form of aggregate results and generalizable statements and the collected data will be confidential (see Podsakoff and Organ, 1986). Consent of the participants, voluntary filling out questionnaires, the confidentiality of identities and responses, and using the collected data only in academic studies eradicate ethical concerns about data collection and use (see Ritchie et al., 2013).

The sampling frame of the study that includes employees working for organizations of different sizes, industries, and capital structures enriched the representativeness of the sample. Although 3,485 employees clicked on the questionnaire’s web address, 854 employees answered the questionnaire (a response rate is 24.5%). Achieving a high response rate is difficult. This particular challenge could be explained by potential respondents’ “declining political and social engagement” and their reluctance to participate due to “increasing number of requests for survey participation” (Breakwell et al., 2020, p. 382). Although using an online questionnaire facilitates access to a representative sample, a lack of contact with units of the sample can explain the non-response rate as well. We removed 15 observations with large numbers of missing data from the study based on the missing data process suggested by Hair et al. (2019). We tested the outliers and the assumptions of normality. Owing to their limited sample size, we excluded the answers of those working in the public sector. We analyzed the data from 792 respondents after the elimination process. This sample size is statistically adequate (Hair et al., 2019) to test our hypotheses. Of the 792 respondents, 311 respondents (39.3%) stated that they “feel a sense of otherness,” while 481 respondents (60.7%) said that they “feel a sense of belonging” in the organization. Approximately 40% of the respondents in both groups were women. The majority of the respondents were in the “28–35-year age range” (36.9%) (the lowest age 22, the highest age 64). 54.7% of the respondents have an associate or bachelor’s degree. 48.6% of those “who feel a sense of otherness” and 56.5% of those “who feel a sense of belonging” were working as “managers” (see Table 1).

**TABLE 1:** Demographic profile of the respondents.

Profile		Sense of otherness		Sense of belonging		Total	
			
		*n*	%	*n*	%	*n*	%
**Gender**							
	Female	124	39,9	188	39,1	312	39,4
	Male	187	60,1	293	60,9	480	60,6
**Education**							
	Associate/bachelor’s degree	170	54,7	263	54,7	433	54,7
	Postgraduate degree	141	45,3	218	45,3	359	45,3
**Position**							
	Manager	151	48,6	272	56,5	423	53,4
	Other	160	51,4	209	43,5	369	46,6
**Age**							
	Less than 28	113	36,3	150	31,2	263	33,2
	28–35	125	40,2	167	34,7	292	36,9
	36 and above	73	23,5	164	34,1	237	29,9
**Length of working time in the organization**							
	Less than 3 years	158	50,8	209	43,5	367	46,3
	3–5 years	93	29,9	133	27,7	226	28,5
	6 years and above	60	19,3	139	28,9	199	25,1
**Total length of working time**							
	5 years	154	49,5	194	40,4	348	44
	6–10 years	75	24,1	100	20,8	175	22,1
	11 years and above	82	26,4	186	38,8	268	33,9

The percentage of respondents working for organizations in the manufacturing sector was 54.3. 45.7% of the respondents were working in the service sector. Employees working in large enterprises were represented with a high percentage (72.1%). A representation of the capital structure of organizations respondents were working for was balanced. 47.1% of them were working in “fully domestic capital” organizations (see Table 2).

**TABLE 2:** Profile of the organizations where respondents work.

Profile		Sense of otherness		Sense of belonging		Total	
			
		*n*	%	*n*	%	*n*	%
**Sector**							
	Manufacturing	182	58,5	248	51,6	430	54,3
	Service	129	41,5	233	48,4	362	45,7
**Size**							
	Large	207	66,6	364	75,7	571	72,1
	Medium–small	104	33,4	117	24,3	221	27,9
**Capital structure**							
	Domestic	169	54,3	204	42,4	373	47,1
	Foreign-owned or joint venture	142	45,7	277	57,6	419	52,9

Considering the channels through which we collect data, the study population consists of relatively qualified employees with associate/bachelor’s degrees. Considering the characteristics such as the profile of the workforce and the distribution of enterprises in Turkey, it can be said that the sample rate of this study is sufficient to represent the population.

### Measures

We used five-point Likert-type scales in this study, as suggested by Hinkin (2005), to collect information about the feelings and opinions of respondents. The Likert-type scale makes it easier to understand and decreases the time to measure constructs (1: strongly disagree, 5: strongly agree). We explained the differences between employees who feel a sense of belonging and those who feel a sense of otherness based on the following four main issues: “competence of diversity actors,” “embeddedness of diversity works in organizational policies/practices,” “diversity awareness in the HRM functions,” and “diversity-related employee satisfaction,” in connection with the hypotheses that we have formulated based on a context that focuses on the Turkish background.

#### Opinions on the competence of diversity actors

Although the middle managers and direct supervisors also have roles, people working in the top management and HRM units of the organizations have the primary responsibility for decisions on and practices of diversity management. Therefore, we focused only on those who work in the top management and HRM units as actors in the diversity issue. The competence of diversity actors was measured with two items (*Cronbach’s alpha* = 0.848). Items measure to what extent they are perceived knowledgeable on diversity management practices (items: Our organization’s top managers have adequate knowledge of managing diversities. HRM employees of our organization have adequate knowledge of managing diversities).

#### Opinions on the embeddedness of diversity works into the organizational policies/practices

To understand the extent to which employees perceive that diversity works are incorporated into business practices, we used eight items (*Cronbach’s alpha* = 0.937) based on prior research (e.g., Pitts, 2006; Choi and Rainey, 2010; Herrera et al., 2011; Sabharwal, 2014) on the embeddedness of diversity (e.g., item: Diversity management practices are integrated into the organizational policies and strategies).

#### Opinions on diversity awareness in the human resource management functions

We measured the opinions on diversity awareness in the HRM functions (recruitment, promotion, training, career development, and appraising performance and compensation) based on three dimensions as follows: (1) justice and impartiality (six items, *Cronbach’s alpha* = 0.919; e.g., *Training procedures and policies are conducted fairly and impartially for all employees in our organization*), (2) sensitivity to the needs of a diverse workforce (six items, *Cronbach’s alpha* = 0.918; e.g., *Training procedures and policies are generated based on the needs of diversified people in our organization*), and (3) equal opportunity (six items, *Cronbach’s alpha* = 0.884; e.g., *Training opportunities for diversified people were restricted in our organization*). These items were adopted from Brinkman’s (1992) items related to recruitment, promotion, and training. One item from the “equal opportunity” dimension was removed to enhance explained variance and make it clearer (omitted item: *Performance of diversified employees was measured based on the same indicators with other employees*).

#### Opinions on diversity-related employee satisfaction

This dimension was measured based on four items (*Cronbach’s alpha* = 0.904). Items question to what extent employees are satisfied with studying in the organization and HRM practices of the organization. Additionally, suggested organization for “acquaintances” and “acquaintances who are diverse” were other questions respondents answered (items: *1*—*In general, I am satisfied with the Human Resource Management activities in the organization I work for; 2—All things considered, I am generally satisfied with working for this organization; 3—I recommend all my acquaintances to work in this organization; and 4—I recommend all my acquaintances who are atypical to work in this organization*).

### Method of analysis and hypothesis tests

Mainly two independent groups of the study, namely, “who feel a sense of belonging” and “who feel a sense of otherness,” were examined whether there was a significant difference between these two independent groups in terms of their opinions about diversity works in their organizations. We compared the opinions of these two groups based on the four dimensions, namely, “competence of diversity actors,” “embeddedness of diversity works into the organizational policies/practices,” “diversity awareness in the HRM functions,” and “diversity-related employee satisfaction,” with a *t*-test.

To summarize the variables with a minimal loss of information and define fundamental dimensions, we performed exploratory factor analysis before testing hypotheses (Hair et al., 2019). We tested the existence of correlations between variables based on the Bartlett sphericity test and the Kaiser–Meyer–Olkin sampling adequacy measure to assess the appropriateness of the factor study, and we did not detect any problems.

## Results

### Opinions on the competence of diversity actors

Those who feel a sense of otherness evaluated the competence of diversity professionals as lower than those who feel a sense of belonging. According to independent *t*-test analysis, the results of Levene’s test for equality of variances showed no violations, *p* = 0.32. The difference between the opinions of the respondents “who feel a sense of otherness (out-group)” (*M* = 2.74, *SD* = 1.08) and “who feel a sense of belonging (in-group)” (*M* = 3.15, *SD* = 1.03) was significant [*t*_(764)_ = 5.29; *p* < 0.001, Cohen’s *d* = 0.43]. So, Hypothesis 1 is supported (see Table 3). A lack of knowledge of those responsible for implementations regarding the scope of diversity works is the possible cause of their inability to address the concerns of people who do not feel a sense of belonging.

**TABLE 3:** Mean comparison of sense of otherness and sense of belonging on perceived diversity works.

	Sense of otherness		Sense of belonging					
			
	*M*	*SD*	*M*	*SD*	*t*	df	*p*	Cohen’*s d*
H1	2.74	1.08	3.15	1.03	5.29	764	0.000	0.43
H2	2.63	0.97	2.85	0.91	2.80	607	0.005	0.23
H3								
H3a)	2.95	1.04	3.42	0.95	6.30	591.86	0.000	0.47
H3b)	3.02	0.95	3.36	0.89	4.82	587.03	0.000	0.37
H3c)	3.05	0.96	3.41	0.85	5.11	558.17	0.000	0.40
H4	3.09	1.07	3.53	0.90	5.87	577.08	0.000	0.44

Cohen’s d = 0.20 small; 0.50 medium; 0.80 large effect size (Cohen, 1992). H1: Competence of diversity professionals. H2: Embeddedness of diversity management into the organizational policies/practices. H3a: Justice and impartiality. H3b: Sensitivity to the needs of people from diverse backgrounds. H3c: Equal opportunity. H4: Diversity-driven organizational satisfaction.

On the contrary, diversity actors who are the formulators and practitioners of diversity works will network with their “in-group” and share more information based on the social identity approaches. This does not happen with those who are in their “out-group.” People who feel a sense of belonging rate the competencies of the diversity actors as adequate.

#### Opinions on the embeddedness of diversity works into the organizational policies/practices

The results of an independent sample *t*-test indicated that Levene’s test for equality of variances showed no violations, *p* = 0.12. Employees “who feel a sense of otherness” assessed diversity works of their organization and harmonization of these practices with the general organizational policies relatively weaker (*M* = 2.63, *SD* = 0.97) than employees “who feel a sense of belonging” (*M* = 2.85, *SD* = 0.91). Hypothesis 2 is supported based on the significance of the difference between the groups [*t*_(607)_ = 2.80; *p* < 0.005, Cohen’s *d* = 0.23]. This finding can be a sign that employees tend to support the decisions and priorities of those in the same group. Decisions and priorities to integrate diversity works into the organizational policies may be raised by employees who feel a sense of belonging. On the contrary, to what extent organizations have a diverse workforce, their attempts will be questioned as long as diversity works are embedded into the organizational policies/practices to meet the demands and priorities of people in the “out-group.”

#### Opinions on diversity awareness in the human resource management functions

Justice and impartiality, sensitivity to the needs of diverse people, and equal opportunity are prominent indicators of diversity awareness in the HRM functions. The results of Levene’s test for equality of variances showed violations, *p* = 0.01. The test statistics of equal variances not assumed showed that employees “who feel a sense of otherness” evaluated justice and impartiality in HRM functions (*M* = 2.95, *SD* = 1.04) less than employees “who feel a sense of belonging” (*M* = 3.42, *SD* = 0.95). The significant difference between the two groups supported Hypothesis 3a [*t*_(591.86)_ = 6.30; *p* < 0.001, Cohen’s *d* = 0.47]. A sensitivity to the needs of people from diverse backgrounds in HRM functions was another dimension. Similarly, the results of Levene’s test for equality of variances showed violations, *p* = 0.03. The test statistics of equal variances not assumed showed a difference between the opinions of employees “who feel a sense of otherness” (*M* = 3.02, *SD* = 0.95) and “who feel a sense of belonging” (*M* = 3.36, *SD* = 0.89). Based on the *t*-test result, Hypothesis 3b is supported [*t*_(587.03)_ = 4.82; *p* < 0.001, Cohen’s *d* = 0.37]. Organizational attempts to provide equal opportunity in HRM functions were perceived relatively lower by those who feel a sense of otherness (*M* = 3.05, *SD* = 0.96) compared with those who feel a sense of belonging (*M* = 3.41, *SD* = 0.85). Hypothesis 3c is supported [*t*_(558.17)_ = 5.11; *p* < 0.001, Cohen’s *d* = 0.40]. The results of Levene’s test for equality of variances showed violations for this dimension, *p* = 0.000. According to the analysis of these three hypotheses, employees who feel a sense of otherness evaluate justice and impartiality, sensitivity to the needs of diverse people, and equal opportunity in HRM functions weaker. Diversity actors who are the formulator and practitioners of diversity works may give more voice to employees who feel a sense of otherness to make the HRM functions comply with the principles such as equality, fairness, and justice.

#### Opinions on diversity-related employee satisfaction

In general, diversity-related satisfaction levels of employees who feel a sense of belonging were higher (*M* = 3.53, *SD* = 0.90) than those of employees who feel a sense of otherness (*M* = 3.09, *SD* = 1.07) in the organization. Because of a significant difference between the satisfaction levels of the two groups, Hypothesis 4 is supported [*t*_(577.08)_ = 5.87; *p* < 0.001, Cohen’s *d* = 0.44]. The results of Levene’s test for equality of variances showed violations, *p* = 0.000. The indifference of individuals to diversity issues in the context of the limited provision of diversity works confronts organizations with the adverse effects of a lack of job satisfaction among employees.

## Discussion

Ethnic and other types of diversity are rapidly becoming visible in organizations as Turkey hosts many migrants, refugees, and displaced persons from the Middle East, Asian, and African countries due to various political and social instabilities (UN News, 2018). This makes Turkey an important context for research on diversity works. In general, various studies (e.g., Küskü et al., 2021; Erdur, 2022) indicate the inadequacy of supportive equality laws and the reliance on organizations to self-manage diversity efforts without accountability structures the apparent absence of supporting discourses such as business case arguments. There are contradictions and paradoxes in diversity works in Turkey. Despite the growing significance of diversity in the Turkish labor market in terms of generic categories of gender, ethnicity, age, disability, and local concerns such as internal and international migration, there is limited provision for diversity works in organizations.

Furthermore, legal and constitutional arrangements for diversity and inclusion remain ceremonial (Özbilgin and Yalkin, 2019) because of the country’s political discourse and policies characterized by nationalism, Islamism, and patriarchal traditionalism (Erdur, 2022). Due to a general atmosphere of indifference and socio-economic turmoil at macro-national and meso-organizational levels, Turkey’s position in terms of gender equality has declined from the first quarter to the last quarter among 150 countries in the previous 20 years (Yamak et al., 2016). As highlighted in previous research (e.g., Küskü and Ataman, 2011), due to the high rate of unemployment and the effect of social and religious values, organizations increasingly prefer recruiting men over women. There remains the challenge for Turkish organizations to develop effective diversity works to garner benefits of diversity such as cognitive, pragmatic, and moral legitimacy (see Özen and Önder, 2021). In particular, it is important for organizations to understand how employees receive diversity works across identity lines.

All four hypotheses, which we formed taking into account the four prominent aspects in the development of diversity works in organizations, were accepted: opinions on (1) competence of diversity actors, (2) embeddedness of diversity works into the organizational policies/practices, (3) diversity awareness in the HRM activities, and (4) diversity-related employee satisfaction. Accordingly, the satisfaction levels of those who feel a sense of otherness in the working environment are lower than those of those who do not feel a sense of otherness. As we outlined above, this result may not be surprising because individuals who feel a sense of otherness are likely to face more challenging circumstances than individuals who feel that they fit in. However, our findings are significant as no previous study has attempted to understand whether the in-groups and out-groups hold different views of diversity works. In the context of a country with adversarial and poorly supported diversity efforts, it is again poignant to see that out-group members are more dissatisfied with diversity works. As a note of caution, we acknowledge that there are ethical problems in enterprises operating in Turkey, such as unfair discrimination among employees, non-promotion, and non-reward of employees according to their abilities (Torlak et al., 2008). These adverse conditions may also help explain why out-groups are more dissatisfied with diversity works in our study.

We controlled organizations’ sector, size, and capital structure where respondents were working to understand whether opinions on four dimensions differ based on these variables. Findings supported the argument that there is no significant difference based on the sector (manufacturing/service). This can be interpreted as expectations and judgments of employees on organizational diversity practices are independent of the sector they are actively working in. The size of organizations produced a significant difference in the “embeddedness of diversity works into the organizational policies/practices.” Diversity work integration was found higher by respondents working in large-sized organizations than in medium-sized organizations. We found that the capital structure of organizations created a significant difference. The competence of diversity actors who are responsible for implementations regarding the scope of diversity works in foreign-owned and joint ventures was perceived as higher. Correspondingly, respondents working in foreign-owned organizations or joint ventures evaluated the embeddedness of diversity works into the organizational policies higher. As expected, their diversity-related employee satisfaction was higher. Additionally, our sample’s lifestyle, culture, education, profession, age, and gender were the most frequently cited diversity dimensions.

Discontent with diversity works occurs at multiple levels, causing a level of cynicism about diversity efforts among workers and scholars alike. Authors such as Dobbin and Kalev (2018) and Noon (2018) have identified the reasons for rising levels of cynicism and dissatisfaction with diversity interventions as an overemphasis on training- and individual-level interventions and a lack of focus on systemic and institutional change in diversity efforts. A recent report (CIPD, 2019) also highlights that apathy and indifference to diversity prevent diversity interventions from being taken up within organizations. Indifference to diversity works presents a major challenge. For example, Kalev et al. (2006) show that those who should be centrally responsible remain indifferent to diversity works, and diversity efforts may be ultimately abandoned. We take this debate further and contribute to an understanding of who might help the individuals who remain cynical and dissatisfied with diversity interventions be. Earlier research on belonging and otherness has highlighted that individuals and groups that feel a sense of belonging have privileged access to resources, opportunities, and networks of power at work (Özbilgin and Woodward, 2004; Neiterman et al., 2015). Yet, we do not know how individuals who feel that they belong and those who feel a sense of otherness evaluate diversity works in organizations.

Social identity approaches imply that, with the effect of stereotyping and depersonalisation, identification with a group changes the individual’s emotions, thoughts, and behaviours (Tajfel and Turner, 1979; Turner et al., 1987). Individuals put group interests above their interests and discriminate against the out-group (Tajfel et al., 1971). Discrimination of out-group members occurs because identification leads individuals to make sense of the world based on in-group values, eliminate uncertainty, and increase their self-esteem as part of a group (Jetten et al., 2017).

As diversity interventions seek to promote social justice and fairness, the out-group members who are more likely to feel excluded experience higher levels of dissatisfaction with the diversity practices, competencies, and policies at work. According to social identity approaches, individuals simultaneously belong to many social categories and some identities sometimes become more important than others. For example, organizational identity can be more important than ethnicity, age, gender, etc. (Hogg and Terry, 2000). Accordingly, diverse people can be combined in a team, and team identification can be enhanced by pro-diversity beliefs (Van Dick et al., 2008). Pro-diversity beliefs decrease identity threat; as a consequence, team members react more positively toward diverse members, and this allows members to work harmoniously, cause creative and quality solutions for tasks, and increase performance (Van Knippenberg et al., 2004), and this also provides positive self-esteem to diverse employees (see Wilkins et al., 2018). Our paper shows how people in an organization cognitively might ignore atypical individuals and unequal practices because of their social identity. We hope that our results may be helpful for organizations to understand this kind of perceptual process to recultivate more pro-diversity beliefs, be aware of apathy and indifference to diverse people, and get advantages from them.

However, our study shows perceptual differences in terms of the effectiveness of diversity works between in-group and out-group members. In-group members show little concern for the effectiveness of diversity works, when diversity works are poorly coordinated and practiced, suggesting the strength of the interplay between social identity and shared concern. Meliou et al. (2021) argue that responsible leadership, i.e., leadership that cares about improving effectiveness of diversity works, emerges out of shared concerns. Thus, our study sheds light on where responsible leaders could draw inspiration for improving diversity works: the opinions of out-group members. Our study calls for attention to differences among workers when shaping diversity works (see Marescaux et al., 2021). To accrue even the much mentioned economic benefits of diversity (Gilbert et al., 1999; Köllen, 2021; Nadarajah et al., 2022), organizations should transcend one size fits all, standardized HRM and diversity works activities for all employees, and co-design HRM practices and diversity works to cater for not only the in-group members but also the out-group members.

## Practical implications

In Turkey, diversity works operate with limited legal protections, low levels of responsibilization of organizations, and poorly formed supportive discourses (Küskü et al., 2021). In this context, our findings highlight that out-group members are dissatisfied with diversity works in organizations. There is a need to move toward more robust legal supports, responsibilization of organizations, and supportive diversity discourses for diversity works to be perceived on equal footing by in-group and out-group members at work. Achievement of this will benefit organizations not only morally but also strategically, such as improving organizational performance (see Siegel, 2020; Showkat and Misra, 2022). Similarly, understanding perceptual differences across social identity lines could help improve HRM processes and career choices and chances of employees (Özbilgin et al., 2005).

The management of organizations should also deal with in-group and out-group differences in assessing the effectiveness of diversity interventions. Specific interventions could be planned to transform the three monkeys of diversity into active supporters. It is of particular import for in-group members to support diversity interventions as allies. Our findings suggest that senior management and managers of HRM units in organizations should not be indifferent to the opinions of employees who feel different. Thus, we call for attention to indifference to difference and how this should be monitored and managed in organizations. Doing this is also essential in terms of sustainability approaches (see Ehnert and Harry, 2012) that emphasize that organizations should also consider their activities’ short- and long-term effects on many stakeholders.

## Limitations and future research directions

Although our study has many important insights to contribute to developing diversity works in business life, naturally, our findings also have some limitations. First, in this study, we did not compare the perceptual differences between out-group and in-group employees based on a particular diversity category (such as age, gender, ethnicity, and race) that caused them to feel different. Instead, we asked about their perception of belonging and otherness work in recognition that sociodemographic characteristics alone may not fully account for in-group and out-group formations. Second, to categorize the participants in terms of belonging and otherness, we have just asked whether they feel a “sense of belonging” or a “sense of otherness.” A question like this is too scarce to attribute an in-group out-group distinction robustly and could be linked with other issues of organizational identification which are pretty distinct from diversity issues. Third, the sample of this research consists of trained, highly skilled/qualified employees.

Future studies could focus on low-qualified workers who are more likely to feel threatened in their positions, bringing different results. Fourth, public sector employees are not included in the research. Private sector organizations, which we focused on, are relatively more dynamic and ready to adapt to external changes (see Tüzüner, 2014). It will be beneficial to conduct similar studies on public sector employees in future studies. Another issue to be careful about when interpreting existing data is the size of the companies the respondents work with. The vast majority of enterprises operating in Turkey are small- and medium-sized enterprises. Even though most of these enterprises try to improve their HRM practices in parallel with economic, social, and technological developments, they have practices reflecting Turkish culture (Tüzüner, 2014). Although it is possible to see the reflection of Western values in managerial practices, imprinting effects of traditional values are still available in Turkish management culture (Aldemir et al., 2003). The fact that the majority (66.6%, see Table 2) of the enterprises in which the people included in the sample of this study work is large-scale organizations. Future studies could focus on small- and medium-sized enterprises, which may bring different results.

The characteristics of the organizational (such as workforce composition and organizational status) and external (such as the impact of the social environment on the social identity process) context affect the practices related to workforce diversity and the perception of these practices by the employees very closely (Joshi and Roh, 2009; Bacouel-Jentjens and Yang, 2019). For this reason, it would not be right to generalize about developing countries based on the results of this study, which was created by collecting data from Turkey. To better understand the grievances arising from the point of view of groups who feel different and to make efforts to eliminate the grievances, we must continue to work toward different contexts.

## Conclusion

In this paper, by collecting data from Turkey, where demographic diversity and concomitant fault lines grow without adequate institutional awareness and effort, we contribute to the debate on the satisfaction and dissatisfaction with diversity works as manifest in perceptual differences between in-group (who feel a sense of belonging) and out-group (who feel a sense of otherness) employees. We demonstrate that the out-group members transcend the three monkeys and express dissatisfaction with diversity issues at work, whereas in-group members are not affected by the absence of attention to diversity works in general. Our empirical contribution is the need for diversity research to focus on in-group and out-group members to explore the varied perceptions of diversity work in organizations.

## Data availability statement

The raw data supporting the conclusions of this article will be made available by the authors, without undue reservation.

## Author contributions

FK and OA contributed to the idea generation, data collection, and preliminary data analysis. All authors list have made a substantial, direct, and intellectual contribution to the work, and approved it for publication.

## Abbreviations

HRM: human resource management.

## References

[B1] Acar F. P. (2010). Analyzing the effects of diversity perceptions and shared leadership on emotional conflict: A dynamic approach. *Int. J. Hum. Resour. Manag.* 21 1733–1753. 10.1080/09585192.2010.500492

[B2] Aldemir C. Arbak Y. Özmen Timurcanday Ö. N. (2003). Türkiye’de İşgörme anlayişi: Tanımı ve boyutlari (The way of doing business in Turkey: Definition and dimensions). *Yönetim Araştırmaları Dergisi* 3 5–28.

[B3] Allen B. J. (2010). *Difference matters: Communicating social identity.* Long Grove, IL: Waveland Press.

[B4] Ashforth B. E. Mael F. (1989). Social identity theory and the organization. *Acad. Manag. J.* 14 20–39. 10.5465/amr.1989.4278999

[B5] Avanzi L. Perinelli E. Bressan M. Balducci C. Lombardi L. Fraccaroli F. (2021). The mediational effect of social support between organizational identification and employees’ health: A three-wave study on the social cure model. *Anxiety Stress Coping* 34 465–478. 10.1080/10615806.2020.1868443 33403860

[B6] Babin B. J. Zikmund W. G. (2015). *Exploring marketing research.* Boston, MA: Cengage Learning.

[B7] Bacouel-Jentjens S. Yang I. (2019). Do we see the same? Discrepant perception of diversity and diversity management within a company. *Employee Relat.* 41 389–404. 10.1108/ER-12-2017-0286

[B8] Benschop Y. (2001). Pride, prejudice and performance: Relations between HRM, diversity and performance. *Int. J. Hum. Resour. Manag.* 12 1166–1181. 10.1080/09585190110068377

[B9] Berger L. J. Essers C. Himi A. (2016). Muslim employees within ‘white’ organizations: The case of Moroccan workers in the Netherlands. *Int. J. Hum. Resour. Manag.* 28 1119–1139. 10.1080/09585192.2016.1166785

[B10] Berrey E. (2014). Breaking glass ceilings, ignoring dirty floors: The culture and class bias of diversity management. *Am. Behav. Sci.* 58 347–370. 10.1177/0002764213503333

[B11] Blalock H. M. (1967). *Toward a theory of minority group relations.* New York, NY: Wiley.

[B12] Bond M. A. Haynes M. C. (2014). Workplace diversity: A social–ecological framework and policy implications. *Soc. Issues Policy Rev.* 8 167–201.

[B13] Breakwell G. Wright D. Barnett J. (2020). *Research methodology in psychology.* London: Sage.

[B14] Brinkman H. S. (1992). *Diversity in the workplace: A review and assessment of the issues that are central to high employee ratings of organizational ability in effectively managing a diverse workforce.* Doctoral dissertation. Denver, CO: The University of Denver.

[B15] Calvard T. (2020). *Critical perspectives on diversity in organizations.* London: Routledge.

[B16] Carrillo Arciniega L. (2021). Selling diversity to white men: How disentangling economics from morality is a racial and gendered performance. *Organization* 28 228–246. 10.1177/1350508420930341

[B17] Carstens J. G. De Kock F. S. (2017). Firm-level diversity management competencies: Development and initial validation of a measure. *Int. J. Hum. Resour. Manag.* 28 2109–2135. 10.1080/09585192.2015.1128460

[B18] Choi S. (2013). Demographic diversity of managers and employee job satisfaction: Empirical analysis of the federal case. *Rev. Public Pers. Adm.* 33 275–298. 10.1177/0734371X12453054

[B19] Choi S. Rainey H. G. (2010). Managing diversity in U.S. federal agencies: Effects of diversity and diversity management on employee perceptions of organizational performance. *Public Adm. Rev.* 70 109–121. 10.1111/j.1540-6210.2009.02115.x

[B20] CIPD (2019). *Diversity management that works: An evidence based view, CIPD research report.* London: CIPD.

[B21] Cohen J. (1992). A power primer. *Psychol. Bull.* 112:155. 10.1037//0033-2909.112.1.155 19565683

[B22] D’Netto B. Shen J. Chelliah J. Monga M. (2014). Human resource diversity management practices in the Australian manufacturing sector. *Int. J. Hum. Resour. Manag.* 25 1243–1266. 10.1080/09585192.2013.826714

[B23] Dang C. T. Volpone S. D. Umphress E. E. (2022). The ethics of diversity ideology: Consequences of leader diversity ideology on ethical leadership perception and organizational citizenship behavior. *J. Appl. Psychol.* Advance online publication. 10.1037/apl0001010 35298214

[B24] Davis P. J. Frolova Y. Callahan W. (2016). Workplace diversity management in Australia: What do managers think and what are organizations doing? *Equal. Divers. Incl.* 35 81–98. 10.1108/EDI-03-2015-0020

[B25] Dineen B. R. Noe R. A. Shaw J. D. Duffy M. K. Wiethoff C. (2007). Level and dispersion of satisfaction in teams: Using foci and social context to explain the satisfaction-absenteeism relationship. *Acad. Manag. J.* 50 623–643. 10.5465/amj.2007.25525987

[B26] Dobbin F. Kalev A. (2018). Why doesn’t diversity training work? The challenge for industry and academia. *Anthropol. Now* 10 48–55. 10.1080/19428200.2018.1493182

[B27] Ehnert I. Harry W. (2012). Recent developments and future prospects on sustainable human resource management: Introduction to the special issue. *Manag. Rev.* 23 221–238.

[B28] Ehrke F. Berthold A. Steffens M. C. (2014). How diversity training can change attitudes: Increasing perceived complexity of superordinate groups to improve intergroup relations. *J. Exp. Soc. Psychol.* 53 193–206. 10.1016/j.jesp.2014.03.013

[B29] Einola K. Alvesson M. (2021). Behind the numbers: Questioning questionnaires. *J. Manag. Inq.* 30 102–114. 10.1177/1056492620938139

[B30] Ely R. J. Thomas D. A. (2001). Cultural diversity at work: The effects of diversity perspectives on work group processes and outcomes. *Adm. Sci. Q.* 46 229–273. 10.2307/2667087

[B31] Erçek M. (2016). Türkiye’de yönetim modalarının firma içi uyarlanma sürecini anlamak: Aktörler, moda etkileşimleri ve çevre ülke konumu (Understanding the in-house adoption process of management fashions in Turkey: Actors, fashion interactions and periphery location). *METU Stud. Dev.* 43 745–777.

[B32] Erdur D. A. (2022). “Diversity and equality in Turkey: An institutional perspective,” in *Research handbook on new frontiers of equality and diversity at work, international perspectives*, eds Klarsfeld A. Knappert L. Kornau A. Ng E. S. Ngunjiri F. W. (Cheltenham: Edward Elgar Publishing Limited), 235–254. 10.4337/9781800888302

[B33] Friday E. Friday S. S. (2003). Managing diversity using a strategic planned change approach. *J. Manag. Dev.* 22 863–880. 10.1108/02621710310505467

[B34] Geare A. Edgar F. McAndrew I. (2006). Employment relationships: Ideology and HRM practice. *Int. J. Hum. Resour. Manag.* 17 1190–1208. 10.1080/09585190600756442

[B35] Geiger K. A. Jordan C. (2014). The role of societal privilege in the definitions and practices of inclusion. *Equal. Divers. Inclus.* 33 261–274. 10.1108/EDI-12-2013-0115

[B36] Gilbert J. A. Ivancevich J. M. Stead B. A. (1999). Diversity management: A new organizational paradigm. *J. Bus. Ethics* 21 61–76.

[B37] Hair J. Black W. Babin B. Anderson R. (2019). *Multivariate data analysis*, 8th Edn. Hampshire: Cengage Learning EMEA.

[B38] Herrera R. Duncan P. A. Green M. Ree M. Skaggs S. L. (2011). The relationship between attitudes toward diversity management in the Southwest USA and the GLOBE study cultural preferences. *Int. J. Hum. Resour. Manag.* 22 2629–2646.

[B39] Hinkin T. R. (2005). “Scale development principles and practices,” in *Research in organizations: Foundations and methods of inquiry*, eds Swanson R. A. Holton E. F. III (San Francisco, CA: Berrett-Koehler Publisher), 161–179.

[B40] Hogg M. A. Terry D. I. (2000). Social identity and self-categorization processes in organizational contexts. *Acad. Manag. Rev.* 25 121–140. 10.5465/amr.2000.2791606

[B41] Jehn K. A. Northcraft G. B. Neale M. A. (1999). Why differences make a difference: A field study of diversity, conflict, and performance in workgroups. *Adm. Sci. Q.* 44 238–251. 10.2307/2667054

[B42] Jetten J. Haslam S. A. Cruwys T. Greenaway K. H. Haslam C. Steffens N. K. (2017). Advancing the social identity approach to health and well-being: Progressing the social cure research agenda. *Eur. J. Soc. Psychol.* 47 789–802. 10.1002/ejsp.2333

[B43] Joshi A. Roh H. (2009). The role of context in work team diversity research: A meta-analytic review. *Acad. Manag. J.* 52 599–627. 10.5465/amj.2009.41331491

[B44] Kalev A. Dobbin F. Kelly E. (2006). Best practices or best guesses? Assessing the efficacy of corporate affirmative action and diversity policies. *Am. Sociol. Rev.* 71 589–617. 10.1177/000312240607100404

[B45] Kamasak R. Özbilgin M. F. Yavuz M. Akalin C. (2019). “Race discrimination at work in the United Kingdom,” in *Race discrimination and management of ethnic diversity and migration at work*, eds Vassilopoulou J. Brabet J. Showunmi V. (Bingley: Emerald Publishing Limited).

[B46] Karsten J. Maznevski M. L. Schneider S. C. (2011). Diversity and its not so diverse literature: An international perspective. *Int. J. Cross Cult. Manag.* 11 35–62. 10.1177/1470595811398798

[B47] Kellough J. E. Naff K. C. (2004). Responding to a wake-up call: An examination of federal agency diversity management programs. *Adm. Soc.* 36 62–90. 10.1177/0095399703257269

[B48] Kemper L. E. Bader A. K. Froese J. (2016). Diversity management in aging societies: A comparative study of Germany and Japan. *Manag. Rev. Spec. Issue* 27 29–49.

[B49] Klarsfeld A. Knappert L. Kornau A. Ngunjiri F. W. Sieben B. (2019). Diversity in under-researched countries: New empirical fields challenging old theories? *Equal. Divers. Inclus.* 38 694–704. 10.1108/EDI-03-2019-0110

[B50] Köllen T. (2021). Diversity management: A critical review and agenda for the future. *J. Manag. Inq.* 30 259–272. 10.1177/1056492619868025

[B51] Kornau A. Knappert L. Erdur D. A. (2021). Advertising, avoiding, disrupting, and tabooing: The discursive construction of diversity subjects in the Turkish context. *Scand. J. Manag.* 37:101151. 10.1016/j.scaman.2021.101151

[B52] Kramar R. Jepsen D. M. (2021). “The diversity context of human resource management,” in *The Oxford handbook of contextual approaches to human resource management*, eds Parry E. Morley M. J. Brewster C. (New York, NY: Oxford University Press).

[B53] Küskü F. (2001). Dimensions of employee satisfaction: A state university example. *METU Stud. Dev.* 28 399–430.

[B54] Küskü F. (2003). Employee satisfaction in higher education: The case of academic and administrative staff in Turkey. *Career Dev. Int.* 8 347–356. 10.1108/13620430310505304

[B55] Küskü F. Ataman B. (2011). Employment interview satisfaction of applicants within the developing context. *Int. J. Hum. Resour. Manag.* 22 2463–2483. 10.1080/09585192.2011.584411

[B56] Küskü F. Araci Ö Özbilgin M. F. (2021). What happens to diversity at work in the context of a toxic triangle? Accounting for the gap between discourses and practices of diversity management. *Hum. Resour. Manag. J.* 31 553–574. 10.1111/1748-8583.12324

[B57] Leach C. W. Van Zomeren M. Zebel S. Vliek M. L. W. Pennekamp S. F. Doosje B. (2008). Group-level self-definition and self-investment: A hierarchical (multicomponent) model of in-group identification. *J. Pers. Soc. Psychol.* 95 144–165. 10.1037/0022-3514.95.1.144 18605857

[B58] Lee E. S. Park T. Y. Koo B. (2015). Identifying organizational identification as a basis for attitudes and behaviors: A meta-analytic review. *Psychol. Bull.* 141:1049. 10.1037/bul0000012 25984729

[B59] Marescaux E. De Winne S. Brebels L. (2021). Putting the pieces together: A review of HR differentiation literature and a multilevel model. *J. Manag.* 47 1564–1595. 10.1177/0149206320987286

[B60] Martins L. L. Milliken F. J. Wiesenfeld B. M. Salgado S. R. (2003). Racioethnic diversity and group members’ experiences- the role of the racioethnic diversity of the organizational context. *Group Organ. Manag.* 28 75–106. 10.1177/1059601102250020

[B61] Meliou E. Ozbilgin M. Edwards T. (2021). How does responsible leadership emerge? An emergentist perspective. *Eur. Manag. Rev.* 18 521–534. 10.1111/emre.12488

[B62] Memon M. A. Salleh R. Mirza M. Z. Cheah J.-H. Ting H. Ahmad M. S. (2021). Satisfaction matters: The relationships between HRM practices, work engagement and turnover intention. *Int. J. Manpower* 42 21–50. 10.1108/IJM-04-2018-0127

[B63] Mergen A. Ozbilgin M. F. (2021). Understanding the followers of toxic leaders: Toxic illusio and personal uncertainty. *Int. J. Manage. Rev.* 23, 45–63. 10.1111/ijmr.12240

[B64] Mor Barak M. E. Lizano E. L. Kim A. Duan L. Rhee M. K. Hsiao H. Y. (2016). The promise of diversity management for climate of inclusion: A state-of-the-art review and meta-analysis. *Hum. Serv. Organ.* 40 305–333. 10.1080/23303131.2016.1138915

[B65] Mullins F. (2018). HR on board! The implications of human resource expertise on boards of directors for diversity management. *Hum. Resour. Manag.* 57 1127–1143. 10.1002/hrm.21896

[B66] Nadarajah S. Atif M. Gull A. A. (2022). State-level culture and workplace diversity policies: Evidence from US firms. *J. Bus. Ethics* 177 443–462. 10.1007/s10551-021-04742-2

[B67] Neiterman E. Salmonsson L. Bourgeault I. L. (2015). Navigating otherness and belonging: A comparative case study of IMGs’ professional integration in Canada and Sweden. *Ephemera Theory Polit. Organ.* 15 773–795.

[B68] Ng E. S. W. Burke R. J. (2005). Personorganization fit and the war for talent: Does diversity management make a difference? *Int. J. Hum. Resour. Manag.* 16 1195–1210. 10.1080/09585190500144038

[B69] Ng E. S. W. Wyrick C. R. (2011). Motivational bases for managing diversity: A model of leadership commitment. *Hum. Resour. Manag. Rev.* 21 368–376. 10.1016/j.hrmr.2011.05.002

[B70] Ng E. S. Sears G. J. Arnold K. A. (2020). Exploring the influence of CEO and chief diversity officers’ relational demography on organizational diversity management: An identity-based perspective. *Manag. Decis.* Epub ahead-of-print. 10.1108/MD-01-2019-0135

[B71] Noon M. (2018). Pointless diversity training: Unconscious bias, new racism and agency. *Work Employ. Soc.* 32 198–209. 10.1177/0950017017719841

[B72] O’Reilly C. A. III Caldwell D. F. Barnett W. P. (1989). Work group demography, social integration, and turnover. *Adm. Sci. Q.* 34 21–37. 10.2307/2392984

[B73] OECD (2020). *All Hands In? Making Diversity Work for All.* Paris: OECD.

[B74] Özbilgin M. F. Woodward D. (2004). ‘Belonging’ and ‘otherness’: Sex equality in banking in Turkey and Britain. *Gender Work Organ.* 11 668–688. 10.1111/j.1468-0432.2004.00254.x

[B75] Özbilgin M. F. Yalkin C. (2019). Hegemonic dividend and workforce diversity: The case of ‘biat’ and meritocracy in nation branding in Turkey. *J. Manag. Organ.* 25 543–553. 10.1017/jmo.2019.39

[B76] Özbilgin M. Küskü F. Erdoğmuş N. (2005). Explaining influences on career ‘choice’: The case of MBA students in comparative perspective. *Int. J. Hum. Resour. Manag.* 16 2000–2028. 10.1080/09585190500314797

[B77] Özbilgin M. Tatli A. Ipek G. Sameer M. (2016). Four approaches to accounting for diversity in global organizations. *Crit. Perspect. Account.* 35 88–99. 10.1016/j.cpa.2015.05.006

[B78] Özen Ş Önder Ç (2021). Diffusion of foreign management practices across Turkish business organizations: A contextualized theory. *Int. Stud. Manag. Organ.* 51 69–92. 10.1080/00208825.2021.1898100

[B79] Palalar Alkan D. Ozbilgin M. Kamasak R. (2022). Social innovation in managing diversity: COVID-19 as a catalyst for change. *Equal. Divers. Incl.* 41, 709–725. 10.1108/EDI-07-2021-0171

[B80] Pitts D. (2009). Diversity management, job satisfaction, and performance: Evidence from US federal agencies. *Public Adm. Rev.* 69 328–338. 10.1111/j.1540-6210.2008.01977.x

[B81] Pitts D. W. (2006). Modeling the impact of diversity management. *Rev. Public Pers. Adm.* 26 245–268. 10.1177/0734371X05278491

[B82] Pitts D. W. Hicklin A. K. Hawes D. P. Melton E. (2010). What drives the implementation of diversity management programs? Evidence from public organizations. *J. Public Adm. Res. Theory* 20 867–886. 10.1093/jopart/mup044

[B83] Podsakoff P. M. Organ D. W. (1986). Self-reports in organizational research: Problems and prospects. *J. Manag.* 12 531–544. 10.1177/014920638601200408

[B84] Point S. Singh V. (2003). Defining and dimensionalising diversity: Evidence from corporate websites across Europe. *Eur. Manag. J.* 21 750–761. 10.1016/j.emj.2003.09.015

[B85] Riccò R. Guerci M. (2014). Diversity challenge: An integrated process to bridge the implementation gap. *Bus. Horiz.* 57 235–245. 10.1016/j.bushor.2013.11.007

[B86] Riketta M. (2005). Organizational identification: A meta-analysis. *J. Vocat. Behav.* 66 358–384. 10.1016/j.jvb.2004.05.005

[B87] Ritchie J. Lewis J. Nicholls C. M. N. Ormston R. (eds). (2013). *Qualitative research practice: A Guide for social science students and researchers.* London: Sage Publications Ltd.

[B88] Roberson Q. M. (2019). Diversity in the workplace: A review, synthesis, and future research agenda. *Annu. Rev. Organ. Psychol. Organ. Behav.* 6 69–88. 10.1146/annurev-orgpsych-012218-015243

[B89] Roberson Q. Perry J. L. (2021). Inclusive leadership in thought and action: A thematic analysis. *Group Organ. Manag.* 47 755–778. 10.1177/10596011211013161

[B90] Saba T. Ozbilgin M. Ng E. Cachat-Rosset G. (2021). Ineffectiveness of diversity management: Lack of knowledge, lack of interest or resistance? *Equal. Divers. Inclus.* 40 765–769.

[B91] Sabharwal M. (2014). Is diversity management sufficient? Organizational inclusion to further performance. *Public Pers. Manag.* 43 197–217. 10.1177/0091026014522202

[B92] Sartori R. Costantini A. Ceschi A. (2022). Psychological assessment in human resource management: Discrepancies between theory and practice and two examples of integration. *Pers. Rev.* 51 284–298.

[B93] Schneider S. K. Northcraft G. B. (1999). Three social dilemmas of workforce diversity in organizations: A social identity perspective. *Hum. Relat.* 52 1445–1467. 10.1023/A:1016976817911

[B94] Shen J. Chanda A. D’Netto B. Monga M. (2009). Managing diversity through human resource management: An international perspective and conceptual framework. *Int. J. Hum. Resour. Manag.* 20 235–251.

[B95] Shore L. M. Chung-Herrera B. G. Dean M. A. Holcombe Ehrhart K. Jung D. I. Randel A. E. (2009). Diversity in organizations: Where are we now and where are we going? *Hum. Resour. Manag. Rev.* 19 117–133. 10.1016/j.hrmr.2008.10.004

[B96] Showkat S. Misra S. (2022). The nexus between diversity management (DM) and organizational performance (OP): Mediating role of cognitive and affective diversity. *Eur. J. Train. Dev.* 46 214–238. 10.1108/EJTD-09-2020-0137

[B97] Siegel D. S. (2020). Why do corporations decide to do good? *J. Econ. Manag. Relig.* 1:2050003. 10.1142/S2737436X2050003X

[B98] Sinicropi S. Cortese D. (2021). (Re) Thinking diversity within sustainable development: A systematic mapping study. *Corp. Soc. Responsib. Environ. Manag.* 28 299–309. 10.1002/csr.2050

[B99] Spector P. E. Brannick M. T. (2009). “Common method variance or measurement bias? The problem and possible solutions,” in *Handbook of organizational research methods*, Vol. 10 eds Buchanan D. A. Bryman A. (Los Angeles, CA: Sage), 346–362.

[B100] Stazyk E. C. Davis R. Liang J. (2012). “Examining the links between workforce diversity, organizational goal clarity, and job satisfaction,” in *Proceedings of the APSA 2012 Annual Meeting Paper*, New Orleans, LA.

[B101] Steffens N. K. Haslam S. A. Schuh S. C. Jetten J. van Dick R. (2017). A meta-analytic review of social identification and health in organizational contexts. *Pers. Soc. Psychol. Rev.* 21, 303–335. 10.1177/1088868316656701 27388779

[B102] Syed J. Ozbilgin M. (2019). *Managing diversity and inclusion: An international perspective.* London: Sage.

[B103] Tajfel H. Turner J. C. (1979). “An integrative theory of intergroup conflict,” in *The social psychology of intergroup relations*, eds Austin W. G. Worchel S. (Monterey, CA: Brooks/Cole), 33–47.

[B104] Tajfel H. Billig M. G. Bundy R. P. Flament C. (1971). Social categorization and intergroup behavior. *Eur. J. Soc. Psychol.* 1 149–178. 10.1002/ejsp.2420010202

[B105] Taras V. Baack D. Caprar D. Dow D. Froese F. Jimenez A. (2019). Diverse effects of diversity: Disaggregating effects of diversity in global virtual teams. *J. Int. Manag.* 25:100689. 10.1016/j.intman.2019.100689

[B106] Tatli A. Özbilgin M. F. (2012). An emic approach to intersectional study of diversity at work: A Bourdieuan framing. *Int. J. Manag. Rev.* 14 180–200. 10.1111/j.1468-2370.2011.00326.x

[B107] Torlak Ö Özdemir Ş Erdemir E. (2008). *İGİAD İş Ahlakı Raporu (IGIAD, Report on Work Ethics).* İstanbul: İGİAD Publishing.

[B108] Triana M. C. Gu P. Chapa O. Richard O. Colella A. (2021). Sixty years of discrimination and diversity research in human resource management: A review with suggestions for future research directions. *Hum. Resour. Manag.* 60 145–204. 10.1002/hrm.22052

[B109] Triandis H. C. (1959). Cognitive similarity and interpersonal communication in industry. *J. Appl. Psychol.* 43 321–326. 10.1037/h0047785

[B110] Tsui A. S. O’Reilly C. A. (1989). Beyond simple demographic effects: The importance of relational demography in superior-subordinate dyads. *Acad. Manag. J.* 32 402–423. 10.5465/256368

[B111] Tsui A. S. Egan T. D. O’Reilly C. A. (1992). Being different: Relational demography and organizational attachment. *Adm. Sci. Q.* 37 549–579. 10.2307/2393472

[B112] Turner J. C. Hogg M. A. Oakes P. J. Reicher S. D. Wetherell M. S. (1987). *‘Rediscovering the social group: A self-categorization theory.* Oxford: Basil Blackwell.

[B113] Tüzüner L. (2014). “Chapter 17–Human resource management in Turkey,” in *The development of human resource management across nations- unity and diversity*, ed. Kaufman B. E. (Cheltenham: Edward Elgar Publishing), 437–460.

[B114] UN News (2018). *Global compact on refugees: How is this different from the migrants’ pact and how will it help?* Available online at: https://news.un.org/en/story/2018/12/1028641 (accessed April 2019).

[B115] Van Dick R. Wagner U. (2002). Social identification among school teachers: Dimensions, foci, and correlates. *Eur. J. Work Organ. Psychol.* 11 129–149. 10.1080/13594320143000889

[B116] Van Dick R. Grojean M. W. Christ O. Wieseke J. (2006). Identity and the extra mile: Relationships between organizational identification and organizational citizenship behavior. *Br. J. Manag.* 17 283–301. 10.1111/j.1467-8551.2006.00520.x

[B117] Van Dick R. Van Knippenberg D. Hägele S. Guillaume Y. R. Brodbeck F. C. (2008). Group diversity and group identification: The moderating role of diversity beliefs. *Hum. Relat.* 61 1463–1492. 10.1177/0018726708095711

[B118] Van Knippenberg D. De Dreu C. K. W. Homan A. C. (2004). Work group diversity and group performance: An integrative model and research agenda. *J. Appl. Psychol.* 89 1008–1022. 10.1037/0021-9010.89.6.1008 15584838

[B119] Vassilopoulou J. April K. Da Rocha J. P. Kyriakidou O. Ozbilgin M. (2016). “Does the ongoing global economic crisis put diversity gains at risk?: Diversity management during hard times – International examples from the usa, south africa, and greece,” in *Handbook of research on race, gender, and the fight for equality*, ed. Prescott J. (Hershey, PA: IGI Global), 424–452. 10.4018/978-1-5225-0047-6.ch019

[B120] Ward A.-K. Beal D. J. Zyphur M. J. Zhang H. Bobko P. (2022). Diversity climate, trust, and turnover intentions: A multilevel dynamic system. *J. Appl. Psychol.* 107 628–649. 10.1037/apl0000923 34110854

[B121] Wilkins S. Butt M. M. Annabi C. A. (2018). The influence of organizational identification on employee attitudes and behaviors in multinational higher education institutions. *J. High. Educ. Policy Manag.* 40 48–66. 10.1080/1360080X.2017.1411060

[B122] Yadav S. Lenka U. (2020). Diversity management: A systematic review. *Equal. Divers. Inclus.* 39 901–929. 10.1108/EDI-07-2019-0197

[B123] Yamak S. Ergur A. Özbilgin M. F. Alakavuklar O. N. (2016). Gender as symbolic capital and violence: The case of corporate elites in Turkey. *Gender Work Organ.* 23 125–146. 10.1111/gwao.12115

[B124] Zhang Y. McGuire S. J. J. (2022). Exploring the mechanism of diversity training through on-the-job embeddedness in a diverse workplace. *Int. J. Train. Res.* 20 14–25. 10.1080/14480220.2021.1934075

